# Impact of age on PUAL as an indicator of opioid effect in adult subjects

**DOI:** 10.1007/s10877-025-01340-9

**Published:** 2025-08-18

**Authors:** Rachel Eshima McKay, Michael A. Kohn, Merlín D. Larson

**Affiliations:** 1 Department of Anesthesia and Perioperative Care, University of California San Francisco (UCSF), 94143-0648 San Francisco, California United States; 2 Department of Epidemiology and Biostatistics, University of California San Francisco (UCSF), San Francisco, California United States

**Keywords:** Quantitative pupil analysis, Opioid pharmacodynamics, Age-related modulation, Opioid induced respiratory depression

## Abstract

Opioid-induced respiratory depression (OIRD) remains a critical safety concern, particularly in older adults, yet timely, reliable detection methods are limited. Decline of pupillary unrest in ambient light (PUAL) has demonstrated potential as a marker of opioid effect in young adult subjects. We evaluated whether previously observed PUAL thresholds for high-risk opioid exposure in younger adults remain valid in 40-60-year-old subjects. Ten healthy volunteers 40–60 years of age underwent PUAL measurement at baseline and every 2.5 min during a 10-minute remifentanil infusion (0.2–0.3 µg/kg/min) and 25-minute recovery period. High-risk opioid exposure was defined primarily by modeled remifentanil effect-site concentration (CEREMI) threshold during infusion. Findings were then combined with previously collected data from 20 younger subjects (aged 20–39 years) undergoing an identical infusion protocol. PUAL declined consistently during infusion and increased toward baseline during recovery (*p* < 0.001). During infusion no significant difference in slope over time or CEREMI was observed between age groups, but during recovery a flatter slope was observed in older subjects (*p* = 0.016). PUAL reliably distinguished between high-versus low-risk opioid exposure during infusion (AUROC = 0.9833 [95% CI: 0.8935, 0.9995]), with interval likelihood ratio (iLR) for high-toxic opioid effect 27.98 (95% CI: 1.79, 438.33) for PUAL < 0.04, 0.75 (95% CI: 0.38, 1.50) for PUAL 0.04-< 0.14, and 0.030 (95% CI: 0.002, 0.477) for PUAL ≥ 0.14. Comparison of discriminatory performance to that of younger subjects showed no significant difference (chi2 = 1.02, *p* = 0.3129). PUAL thresholds for high-risk opioid exposure are consistent between younger and older adults and do not require age-specific adjustment up to age 60. PUAL offers a reliable, real-time marker of opioid effect with potential to enhance early OIRD detection in adults.

**Supplementary Information:**

The online version contains supplementary material available at 10.1007/s10877-025-01340-9.

## Introduction

 Opioid-induced respiratory depression (OIRD) remains a leading cause of avoidable harm in hospitalized patients and a significant contributor to perioperative morbidity and mortality [1]. Despite the growing use of multimodal analgesia, opioids remain essential for managing acute pain [2, 3]. OIRD is notoriously difficult to detect at early stages, and clinical signs may be absent or unrecognized until patient harm occurs [4]. 

Pupillary unrest in ambient light (PUAL), refers to the spontaneous, bilateral oscillation of pupil diameter observed in awake healthy subjects, and is thought to originate centrally from phasic inhibitory impulses targeting the Edinger-Westphal nucleus. PUAL declines consistently with increasing opioid exposure in young adult subjects, presumably due to opioid-induced suppression of this phasic inhibitory activity and for that reason, the measure has been investigated recently as a possible indicator of central opioid effect. Preliminary data suggest PUAL decline may be a promising non-invasive marker of opioid toxicity. In a prior study of healthy adults aged 20–39, remifentanil produced dose-dependent reduction and eventual loss of PUAL, corresponding with high opioid concentrations and episodes of hypoxemia, even while subjects remained conscious [5]. Specific PUAL thresholds (< 0.04 and ≥ 0.14) accurately distinguished high-toxic from low-to-moderate opioid exposure defined by experimental time-points, with an AUROC of 0.9566 (95% CI: 0.9244, 0.9798) [6]. 

Age-related decline in central Edinger Westphal inhibition may alter PUAL’s predictive value as an indicator of opioid exposure in older adults. Irene Loewenfeld’s classic study of dark-adapted pupils found pupil size declines by 0.4 mm per decade between ages 20 and 60, a trend attributed to age-related loss of central inhibition [7]. Subsequent studies confirmed this relationship across a variety of lighting conditions [8] showing that both PUAL and pupil size decline with age in unmedicated adults studied under ambient light [9, 10]. 

In this study, we investigated whether the previously established PUAL thresholds for opioid risk remain valid in adults aged 40–60, or whether age-related changes in baseline pupil dynamics necessitate modified thresholds. Using the same remifentanil infusion protocol as our prior study, we assessed whether PUAL preserves its discriminatory accuracy in older adults- a question of clinical relevance given the heightened opioid sensitivity observed in elderly patients [11].

## Methods

Following approval by the UCSF Committee on Human Research (NCT05391555), we recruited healthy adult volunteers aged 40–60 years to participate in a study examining pupillary measurements taken before, during and after an infusion of remifentanil. Written informed consent was obtained from all participants.

After an 8-hour fasting period, subjects underwent peripheral IV placement and received aprepitant and ondansetron for antiemetic prophylaxis. Pupillary unrest in ambient light (PUAL) was recorded at baseline, and every 2.5 min during a 10-minute remifentanil infusion (0.2 µg/kg/min for 5 min followed by 0.3 µg/kg/min for 5 min) and 25-minute recovery period, with final measurement taken 15 min later, 50 min after start of the infusion. The infusion protocol was designed to achieve a broad range of estimated remifentanil effect-site concentrations (CEREMI), based on the Minto pharmacokinetic model, with peak levels in the 4–6 ng/mL range previously associated with significant anesthetic effect and a high probability of apnea [12–14]. All studies were conducted in the UCSF Hypoxia Laboratory under ambient light conditions (~ 200 lx).

Pupillary measurements were obtained using a handheld infrared pupillometer (Neuroptics PLR-3000). Subjects looked into a dark, cone-shaped eyepiece with the left eye while the contralateral eye was shielded by the operator’s hand. A 50 µW light source (~ 350 lx) was directed into the measured eye, and a 10-second infrared video was captured. These videos were post-processed using fast Fourier transformation to generate the PUAL value. Calibration against known apertures (2.6–4.8 mm) enabled subtraction of background noise and definition of the lower boundary of the PUAL scale [15]. Incidence and timing of oxyhemoglobin desaturation (SpO_2_ < 90%) were recorded, with prompt administration of supplemental oxygen to restore saturation to ≥ 95%.

The primary study objective was to examine the relationship between PUAL and remifentanil exposure, both during infusion (0–10 min) and recovery (10–35 min), and to assess PUAL’s ability to discriminate between low- versus high-risk opioid effect states. Data from these newly recruited older adult subjects were combined with data from a previously studied cohort of younger adults (20–39 years). Analyses were initially performed using time-points relative to start of the infusion as the exposure variable due to its intuitive interpretability and prior use in our earlier work, and outcomes were compared in the dichotomized age groups. To account for age-related decline in drug distribution and clearance we subsequently used estimated remifentanil effect-site concentration (CEREMI), calculated from the Minto model, as the exposure variable, with outcomes compared by both continuous age and dichotomous age groups.

We used linear mixed-effects models to evaluate the relationship between PUAL and opioid exposure (time or CEREMI), including age (grouped or continuous) and interaction terms. Models included subject-level random intercepts and slopes to account for between-subject variability in baseline PUAL and response trajectory. Likelihood ratio tests assessed whether age modified the slope of the exposure-response relationship and margins commands were used to visualize predicted PUAL values with 95% confidence intervals at defined exposure levels. During the recovery phase, analyses were restricted to the 10–35-minute interval to reflect the pharmacodynamically active window. At later time points, CEREMI values were negligible, and PUAL had plateaued toward baseline, potentially obscuring slope estimates.

For dichotomous outcomes- apnea with desaturation < 90%, and ≥ 90% suppression of baseline PUAL-Cox proportional hazards models were used to compare incidence and time to onset. CEREMI and PUAL were also tested as predictors in place of time, stratified by age group. Received operating characteristic (ROC) analysis was employed to evaluate PUAL’s ability to distinguish between high-risk versus low-risk opioid exposure. High-risk state was defined primarily by modeled CEREMI ≥ 2.0 ng/mL (vs. <2.0 ng/mL) during infusion, and secondarily by the 5.0–12.5-minute (vs. 0–2.5 min) time window from start of the infusion. AUROC was calculated using the DeLong method. Interval likelihood ratios (iLRs) were estimated using previously defined PUAL cutoffs (0-< 0.04: high risk; ≥ 0.04-< 0.14: indeterminant risk; ≥ 0.14: low risk), with Haldane–Anscombe correction applied to zero-cell estimates to avoid division by zero. Final analyses included pooled data from both age groups. In contrast to younger participants, who completed both interactive and non-interactive protocols, older participants completed a single non-interactive infusion-recovery sequence.

## Results

### Participants

Ten healthy volunteers aged 40–60 years completed a 10-minute remifentanil infusion followed by a 25-minute recovery period, with characteristics (mean [range] or count) age 48.7 years [41–60], male/female 5/5, height 166.7 cm [152.4 − 180.3], weight 66.4 kg [51.4–75.9], and BMI 23.9 kg/m2 [20.1–26.7]. Self-identified race/ethnicity were Asian (*n* = 3), White (*n* = 4), Latino (*n* = 2), and Black (*n* = 1). Characteristics of twenty previously studied participants aged 20–39 included age 25.6 [20–32], male/female 7/13, height 166.8 cm [154.9–188.0], weight 64.7 kg [50.0–95.5], BMI 23.2 kg/m2 [20.0–35.0]. Self-identified race/ethnicity were Asian (*n* = 13), White (*n* = 5) and Latino (*n* = 2).

### PUAL and estimated remifentanil concentration over time

**Fig. 1:**
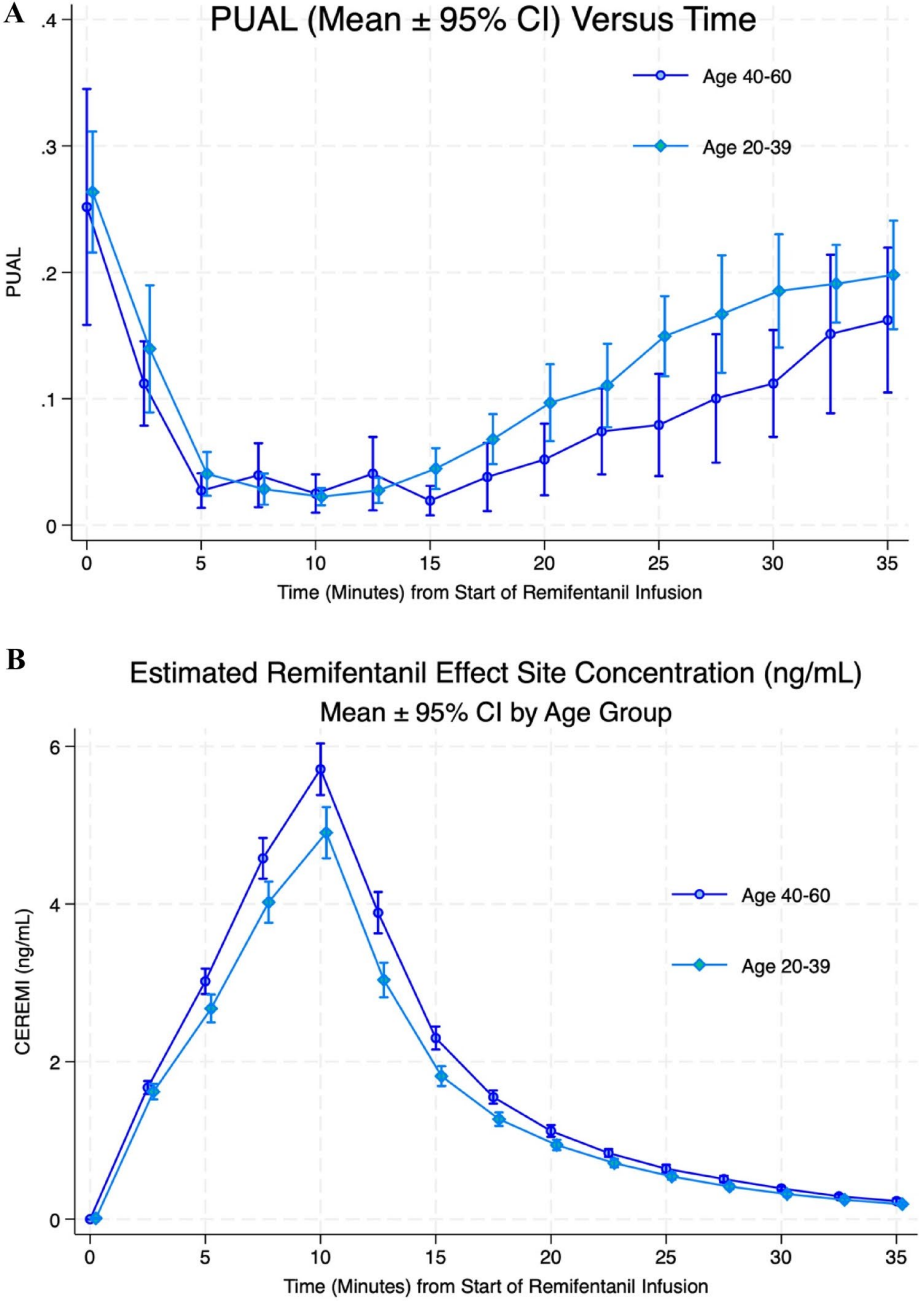
**A** Pupillary unrest (PUAL) values (mean ± 95% confidence interval) over time during the 10-minute remifentanil infusion and 25-minute recovery period are shown, in older and younger age groups. **B** Estimated remifentanil effect site concentrations (CEREMI, mean ± 95% CI, ng/mL) during the 10-minute infusion and 25-minute recovery are shown. The age-dependent lower rate of modeled drug clearance from the central compartment, in the context of a non-age-adjusted infusion scheme, explains the higher estimated drug concentration observed in the older subjects (5.71 ± 0.46 vs. 4.91 ± 0.69 ng/mL, *p* = 0.0025)

PUAL and modeled remifentanil effect site concentration (means ± 95% confidence intervals) over time are shown in Fig. 1A and B. Remifentanil was administered to each subject at a weight-based infusion rate (total body weight), without adjustment for age, height or sex. Estimated effect site concentrations (CEREMI) were calculated using the Minto pharmacokinetic model implemented in the TivaTrainerX application and included in the analysis for both infusion and recovery periods.

As a reflection of age-adjusted decline in drug clearance and volume of distribution within the pharmacokinetic model, older subjects exhibited significantly higher average peak CEREMI values at 10 min compared to younger subjects (5.71 ± 0.46 vs. 4.91 ± 0.69 ng/mL, *p* = 0.0025), as well as a flatter CEREMI-versus-time slope during infusion (0.49 vs. 0.57 ng/mL/min; difference = − 0.08, Wald χ² = 11.48, *p* = 0.0007). To account for these age-related differences, both time and CEREMI have been included as covariates in subsequent models.

### Effect of age across time: infusion (0–10 min)

During the remifentanil infusion, PUAL declined significantly over time in all participants. In a mixed-effects model using elapsed time as a predictor, the average slope of PUAL change was − 0.0210 units/minute (95% CI: − 0.0270, − 0.0151) in older subjects versus − 0.0237 units/minute (95% CI: − 0.0279, − 0.0195) in younger subjects, with interaction between age group and time not statistically significant (difference in slope = − 0.0027, *P* = 0.471; Wald χ² = 0.52, *p* = 0.4711), indicating similar rates of PUAL suppression in both age groups during infusion (Figure S1A). Across all 20-60-year-old subjects, the interaction between age and time was not statistically significant (β = 0.0002 per minute-year; *p* = 0.191), suggesting no discernable difference in opioid-induced PUAL suppression across the age spectrum. Predicted PUAL trajectories by decade are shown in Figure S1B.

### Effect of age across modeled remifentanil effect site concentration (CEREMI): infusion

PUAL declined significantly with increasing remifentanil effect-site concentration (CEREMI) in both age groups, by an average of − 0.0474 units/ng/mL (95% CI: − 0.0558, − 0.0390) in younger participants versus − 0.0370 units/ng/mL (95% CI: − 0.0473, − 0.0267) in older participants. The difference in slopes between groups was not statistically significant (slope difference = − 0.0104; Wald χ² = 2.35, *P* = 0.1251, Figure S1C). Among all 30 subjects, a statistically significant interaction between age and CEREMI on PUAL was observed during infusion (Wald χ² = 5.93, *P* = 0.014), suggesting that the suppressive effect of CEREMI on PUAL was modestly attenuated by increasing age (Figure S1D).

### Effect of age across time: recovery (10–35 min)

During recovery, PUAL increased over time in all participants, with a significantly steeper slope in younger participants (0.0080 units/minute; 95% CI: 0.0069, 0.0091) compared to older participants (0.0057 units/minute; 95% CI: 0.0041, 0.0072). This interaction between age group and time was statistically significant (difference in slope = 0.0023, *P* = 0.016; Wald χ² = 5.81, *p* = 0.0159), consistent with more rapid physiologic recovery in younger individuals (Figure S2A). Across all 30 subjects, PUAL increased significantly over time (slope = 0.0109 units/minute; 95% CI: 0.0079, 0.0138; *P* < 0.001), with a significant interaction between age and time (β = − 0.0001 per minute-year; 95% CI: − 0.0002, − 0.0000; *P* = 0.018), confirming slower PUAL recovery with increasing age (Figure S2B).

### Effect of age across modeled remifentanil concentration (CEREMI): recovery (10–35 min)

As CEREMI declined during recovery, a significant age-group-related difference in the slope of PUAL recovery was observed, with 0.0636 units/ng/mL (95% CI: 0.0522, 0.0750) in younger versus 0.0309 units/ng/mL (95% CI: 0.0168, 0.0449) in older participants (difference = − 0.0327; *P* < 0.001; Wald χ² = 12.55, *P* = 0.0004; Figure S2C). Among all 30 subjects, declining CEREMI was associated with increasing PUAL (β = − 0.037; *P* = 0.014), with a marginally significant interaction (β = 0.0008; *P* = 0.050) again suggesting that the suppressive effect of CEREMI on PUAL was attenuated by increasing age. The slope of PUAL versus CEREMI was modeled as − 0.037 + 0.0008 × age, flattening from − 0.053 at age 20 to approximately − 0.021 at age 60 (Figure S2D).

### Time-to-event conditions by age

#### PUAL at time of first desaturation to SpO_2_ < 90%

**Fig. 2:**
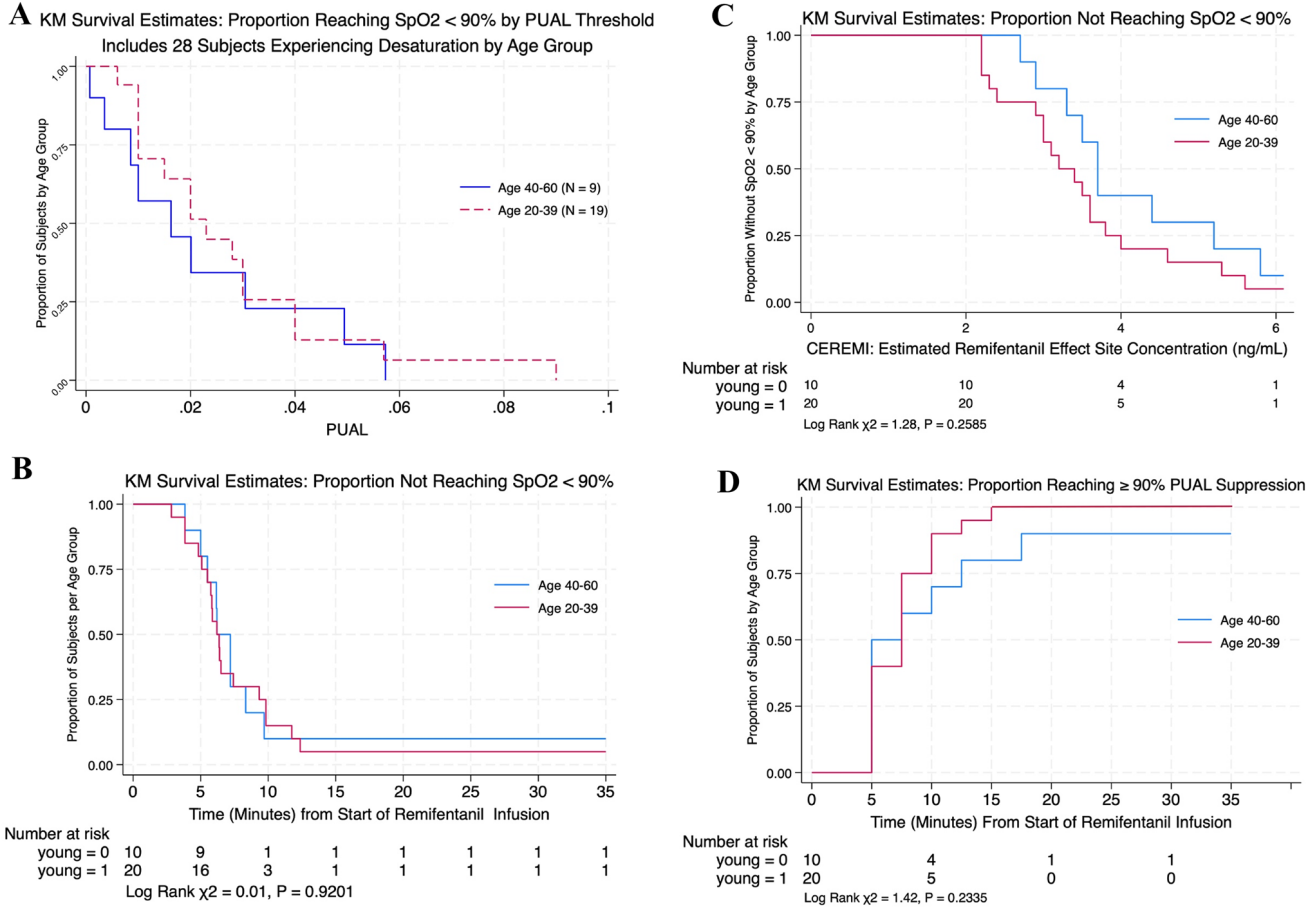
**A** Cumulative proportion of subjects reaching SpO_2_ < 90% as a function of PUAL, stratified by age group. Only participants experiencing desaturation (*n* = 28) are included. No significant difference was observed in the distribution of PUAL at desaturation between age groups (Wilcoxon rank-sum test, exact *p* = 0.9900). **B** Kaplan Meier survival curve showing the cumulative proportion of participants without oxyhemoglobin desaturation (SpO_2_ ≥ 90%) as a function of time, in older and younger age groups. The time-to-event did not differ statistically by age group, occurring at median (95% CI) 6.2 [3.8, 8.3] minutes vs. 6.2 [5.1, 9.3] minutes in the younger subjects (log-rank χ^2^ = 0.01, *P* = 0.9201). Number at risk indicates the number of participants at each time point who had not yet experienced desaturation. **C** Kaplan-Meier curves showing the cumulative proportion of participants without oxyhemoglobin desaturation (SpO_2_ ≥ 90%) as a function of estimated remifentanil effect-site concentration (CEREMI), with subjects stratified by age group. Desaturation events occurred at median (95% CI) CEREMI = 3.7 (2.7, 5.2) in older subjects versus 3.2 (2.4, 3.8) lower CEREMI levels in the younger subjects (log-rank χ^2^ = 1.28, *P* = 0.26). Number at risk indicates the number of participants at each CEREMI level who had not yet experienced desaturation. **D** Kaplan–Meier survival curves showing the cumulative proportion of participants reaching ≥ 90% suppression of pupillary unrest (PUAL) over time following start of the remifentanil infusion, stratified by age group. Median time to suppression did not differ between younger and older participants (log-rank χ^2^ = 1.42, *p* = 0.2335)

In all participants, time (median [IQR]) from start of the infusion to first SpO_2_ < 90% was 6.2 [5.0, 8.2] minutes. A total of 19/20 younger and 9/10 older subjects experienced oxyhemoglobin desaturation (SpO_2_ < 90%) during the experiments. The median PUAL at the time of desaturation was 0.027 (IQR: 0.017–0.038) in the younger group and 0.030 (IQR: 0.020–0.040) in the older group (Wilcoxon rank-sum test, exact *p* = 0.9900). One subject in each group did not desaturate, with the lowest observed PUAL values in these subjects being 0.006 (younger) and 0.010 (older), indicating that in those two individuals, desaturation did not occur even at relatively suppressed levels of PUAL (Fig. 2A).

#### Time to desaturation

The time to oxyhemoglobin desaturation (SpO_2_< 90%) was evaluated using Kaplan–Meier survival analysis, stratified by age group. Desaturation occurred in all cases prior to 12.5 min, with no significant difference in time to event among younger versus older subjects. The median time to SpO_2_ < 90% was 6.2 min in both groups (log rank χ^2^ = 0.01, *p* = 0.9201; see Fig. 2B).

#### Estimated remifentanil effect site concentration (CEREMI) at desaturation

While the younger group desaturated at lower CEREMI values than the older group (at median [range] 3.2 [2.2, 5.6] vs. 3.7 [2.7, 5.8] ng/mL), the Kaplan-Meyer analysis showed this difference to be non-statistically significant (log-rank χ² = 1.28, *P* = 0.2585, Fig. 2C). These findings suggest that the relationships between time and estimated effect-site concentration versus risk of desaturation did not differ substantially by age group under the conditions studied (Fig. 2C).

#### Time to ≥ 90% PUAL suppression

PUAL decline from baseline was significant in all subjects, with 30/30 showing ≥ 80% decline and 29/30 showing ≥ 90% decline after start of the infusion compared to the baseline measure. Time to first reach ≥ 90% PUAL suppression did not differ significantly between age groups- with median time 7.5 min (IQR: 5.0, 8.75) in the younger group and 6.25 min (IQR: 5.0, 12.5) in the older group. Note that PUAL was assessed at 2.5-minute intervals; thus, summary statistics reflect interpolated values from Kaplan–Meier survival estimates rather than directly observed timepoints (log-rank χ² = 1.42, *p* = 0.2335; see Fig. 2D).

### Detection of high-risk opioid exposure

**Fig. 3:**
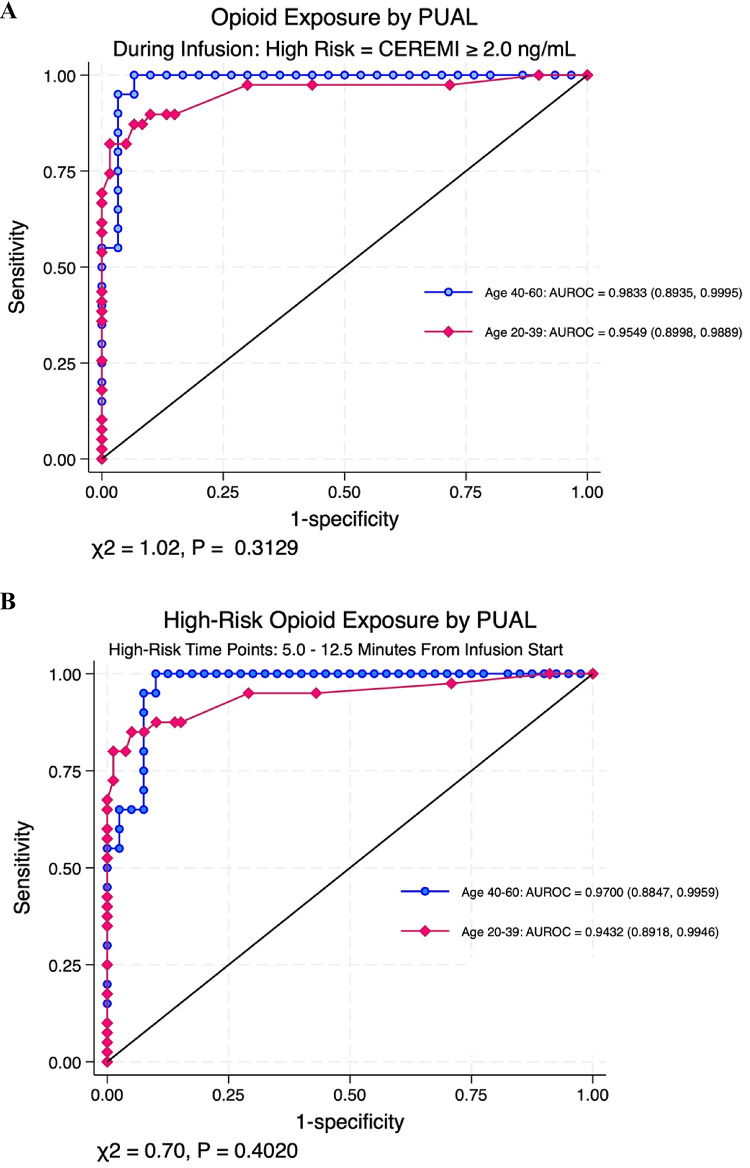
**A** PUAL (pupillary unrest) distinguished between high-risk opioid exposure defined as CEREMI (estimated remifentanil effect site concentration) ≥ 2.0 ng/mL versus low-moderate-risk opioid exposure versus CEREMI (estimated remifentanil effect site concentration) < 2.0 ng/mL with excellent discrimination (AUROC = 0.9833 [0.8935, 0.9995] in older subjects and 0.9549 [0.8998, 0.9889] in younger subjects), difference by age group not statistically significant (χ2 = 1.02, *P* = 0.3129). **B**: PUAL (pupillary unrest) distinguished between high-toxic opioid exposure (time window 5.0–12.5 min post start of the remifentanil infusion) versus low-moderate opioid exposure (time window 0–2.5 min post start of remifentanil infusion) with excellent discrimination (AUROC = 0.9700 [0.8847, 0.9959] in older subjects and 0.9432 [0.8918, 0.9946] in younger subjects), difference by age group not statistically significant (χ2 = 0.70, *P* = 0.4020)

Using estimated effect-site remifentanil concentration (CEREMI), we next evaluated PUAL’s ability to distinguish high-risk opioid exposure defined as CEREMI ≥ 2.0 ng/mL during the infusion phase (0–10 min), with AUROC 0.9833 (95% CI: 0.8935, 0.9995) in older subjects compared to 0.9549 (95% CI: 0.8998, 0.9889) in younger subjects (chi2 for difference = 1.02, *p* = 0.3129, Fig. 3A). The interval likelihood ratios (iLRs) for high opioid exposure based on CEREMI ≥ 2.0 in the older versus younger subjects during infusion were 27.984 (1.787, 438.327) and 26.852 (3.851, 187.238) for PUAL < 0.04 (high risk range), 0.754 (0.380, 1.497) and 1.046 (0.555, 1.971)) for PUAL 0.04-< 0.14 (indeterminant range), and 0.030 (0.002, 0.477) and 0.024 (0.003, 0.167) for PUAL ≥ 0.14 (low risk range). Additional details including data using experimental time points as an alternative risk covariate and all phases of the experiment in combined 30 subjects are included in Table S1.

Utilizing the entire observation period (0–35 min) in all 30 subjects, PUAL distinguished CEREMI ≥ 2.0 ng/mL with an AUROC of 0.9034 (95% CI: 0.8757, 0.9311). Interval likelihood ratios were 5.87 (95% CI: 4.26, 8.09) for PUAL < 0.04, 0.633 (95% CI: 0.476, 0.843) for PUAL 0.04 - < 0.14, and 0.009 (95% CI: 0.001, 0.147) for PUAL ≥ 0.14. These findings support the generalizability of PUAL thresholds for identifying high- versus low-risk opioid conditions based on modeled opioid concentration (see supplemental Table S1 for details).

Time-to-event analysis confirmed that episodes of oxygen desaturation (SpO_2_ < 90%) occurred most frequently between 5.0 and 12.5 min following the start of remifentanil infusion. Based on that distribution, time points within this window were operationally defined as high-risk for opioid toxicity, while early time points at 0 and 2.5 min, prior to the onset of any desaturation events, were defined as low risk. Among the 40-60-year-old subjects, using these time-based risk windows as an alternative exposure axis, PUAL demonstrated excellent discrimination between high- and low-to-moderate risk opioid exposure, with an AUROC of 0.9700 (95% CI: 0.8847, 0.9959). All PUAL values < 0.04 occurred during the high-risk time windows, yielding an interval likelihood ratio (iLR) of ∞ (95% CI: 4.65 to ∞), given perfect agreement within sampling limits. To account for cells containing zeros in the contingency table, the Haldane-Anscombe continuity correction was applied, producing a corrected iLR of 25.82 (95% CI: 1.65–402.99) [16, 17]. In contrast, PUAL values ≥ 0.14 were rarely observed during high-risk time points and were strongly associated with low opioid effect, yielding a corrected iLR of 0.022 (95% CI: 0.012, 0.561). Intermediate PUAL values between 0.04 and < 0.14 were indeterminant (iLR = 0.83 [95% CI: 0.45, 1.52]). In younger subjects (ages 20–39), performance was similar, with AUROC 0.9566 (95% CI: 0.9244, 0.9798), and PUAL values < 0.04 conferring an iLR of 14.59 (95% CI: 5.59, 38.10) for high-risk opioid exposure (Table 1; Fig. 3B).

**Table 1:** Interval likelihood ratios (iLRs) for high-risk opioid exposure, defined either by time (5.0–12.5 min after infusion start) or by estimated remifentanil concentration (CEREMI) ≥ 2.0 ng/ml. PUAL (pupillary unrest) values < 0.04 indicate intense, high-risk opioid effect; ≥ 0.14 suggest low-risk opioid effect; 0.04-<0.14 values were indeterminant

PUAL Range	iLR (95% CI): Time Based Threshold (5.0–12.5 min)		iLR (95% CI): CEREMI during Infusion (≥ 2.0 ng/mL)	
**< 0.04**	*All*:	**17.8** (6.8–46.8)	*All*:	**40.2** (5.7–282.1)
	*Older*:	**25.8** (1.7–403.0)	*Older*:	**28.0** (1.8–438.3)
	*Younger*:	**14.6** (5.6–38.1)	*Younger*:	**26.9** (3.9–187.2)
**0.04 - < 0.14**	*All*:	0.92 (0.64–1.33)	*All*:	0.91 (0.57–1.45)
	*Older*:	0.83 (0.45–1.52)	*Older*:	0.75 (0.38–1.50)
	*Younger*:	0.94 (0.61–1.45)	*Younger*:	1.05 (0.56–1.97)
**≥ 0.14**	*All*:	**0.004** (0–0.064)	*All*:	**0.017** (0.002–0.121)
	*Older*:	**0.022** (0.012–0.561)	*Older*:	**0.030** (0.002–0.477)
	*Younger*:	**0.005** (0–0.075)	*Younger*:	**0.024** (0.003–0.167)

## Discussion

In this cohort of healthy 40-60-year-old adults, PUAL values < 0.04 reliably identified high-toxic opioid exposure during a standardized remifentanil infusion protocol. PUAL declined consistently as modeled remifentanil effect-site concentration rose during infusion and recovered as concentrations fell, supporting its value as a dynamic marker of opioid effect. Restoration of PUAL toward baseline occurred more slowly in older subjects during the post-infusion period, possibly reflecting age-dependent decline in the rate of drug clearance, slower physiologic recovery, or both.

Comparison of findings in older adults to our prior study of younger adults (aged 20–39) demonstrated similar discriminative performance of PUAL to detect opioid effect. Discrimination of high-risk opioid effect by PUAL showed no clear or significant age-related interaction regardless of defining criteria (χ^2^ = 1.02, *p* = 0.3129 using CEREMI ≥ 2.0 ng/mL during infusion, χ² = 0.33, *p* = 0.5645 using time points 5–12.5 min from start of infusion, and χ2 = 2.61, *p* = 0.1061 using CEREMI ≥ 2.0 ng/mL across the entire period of observation). Although numerous factors encountered during typical patient care, including chronic illness, autonomic impairment, dynamic pain level, and fluctuating environmental stimulation may confound the significance of pupillary measures and/or transiently obscure signs of opioid toxicity, PUAL appears to be a consistent and independent physiologic indicator of opioid effect in healthy subjects within our experimental setting [5, 18]. 

The overall pattern of opioid-PUAL response was preserved across adults 20–60 years of age. However, a broader age range or larger sample might have uncovered more substantial age-related differences. Our data indicated a modest suppressive effect of age on PUAL decline with rising opioid concentration. A recent observational study of 120 healthy adults found a small but significant age-related decline in resting PUAL among subjects 18 - >70 years of age,^10^ although the extent to which such differences in baseline PUAL might predict altered opioid-PUAL dynamics remains an unproven question.

We suggest that previously established PUAL thresholds for detecting opioid toxicity remain valid in adults up to at least age 60, finding no evidence at this time that age-specific adjustment in the studied age-range is needed. Given the heightened susceptibility of older adults to opioid-induced respiratory depression, early detection remains a critical priority. PUAL monitoring may offer an efficient, noninvasive method for identifying high-risk opioid states and enhancing safety when used alongside traditional physiologic and behavioral measures.

## Limitations

This study has several limitations. First, while our aim was to evaluate the validity of previously established PUAL thresholds in older adults, our sample was small and included participants only up to age 60. We acknowledge that this range precludes detection of more profound physiologic change that may exist or emerge later in life; our choice of a conservative upper age limit was driven by ethical and practical constraints related to the safety of reasonableness of subjecting frail geriatric volunteers to hypoxia and other adverse sequelae of opioid toxicity. Second, remifentanil was administered using total body weight–based dosing without age or LBW adjustment, consistent with common U.S. clinical practice. Although post-hoc CEREMI modeling allowed for individualized pharmacokinetic estimates, our approach resulted in relatively higher modeled effect-site concentrations to our older subjects, potentially introducing a modest exposure bias that we acknowledge. Future studies including more diverse age ranges, alternative dosing strategies, and clinical populations are needed to fully define the applicability of PUAL across the aging spectrum.

## Supplementary Information

Below is the link to the electronic supplementary material.

Supplementary Material 1

## Data Availability

No datasets were generated or analysed during the current study.
