# Supplementary material for: Impact of age on PUAL as an indicator of opioid effect in adult subjects
Source: J Clin Monit Comput. 2025 Aug 18;40(2):479–87. doi: 10.1007/s10877-025-01340-9 (PMC13053361; doi:10.1007/s10877-025-01340-9)
Supplement: Supplementary file 1 — Supplementary Material 1 [file 10877_2025_1340_MOESM1_ESM.docx]

**Supplemental Materials**


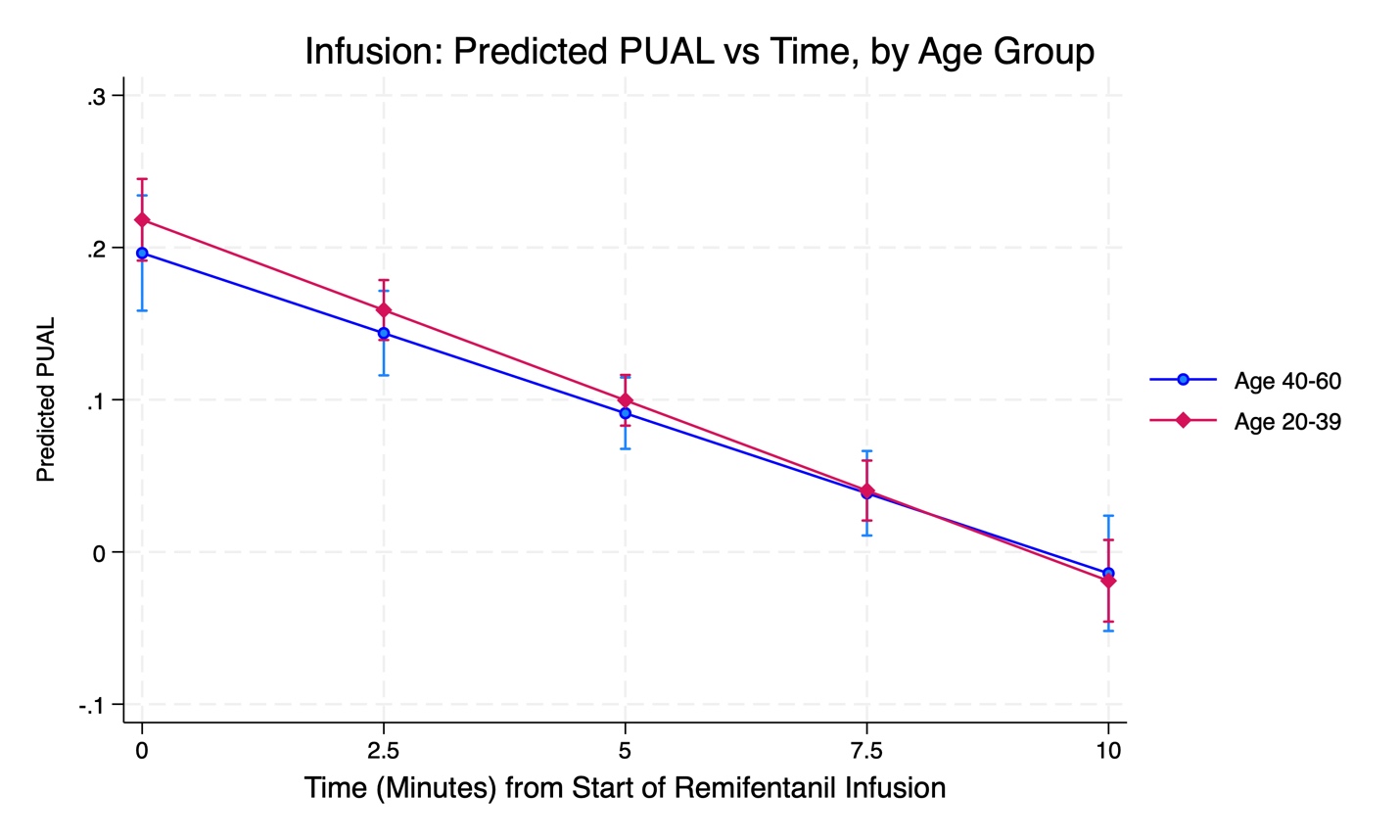


**Figure S1A.** Pupillary unrest (PUAL) declined progressively over time during the infusion, with no significant difference in the rate of decline observed between age groups. Average predicted PUAL ± 95% CI shown.

**
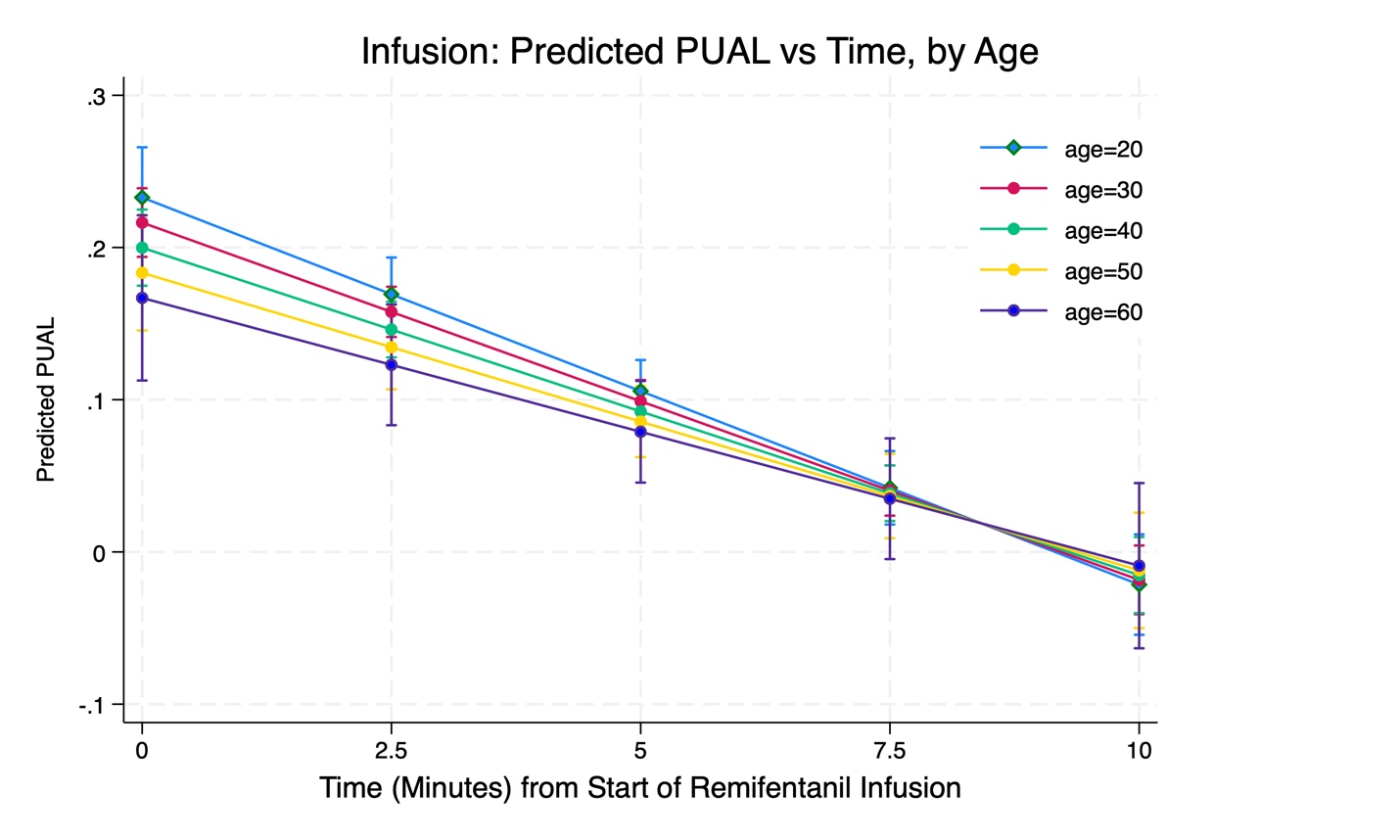
**

**Figure S1B.** Pupillary unrest (PUAL) declined significantly during infusion across all participant ages, with no statistically significant age-related difference in suppression slope (difference -0.0002 units per minute*year, P = 0.191). Average predicted PUAL ± 95% CI shown.


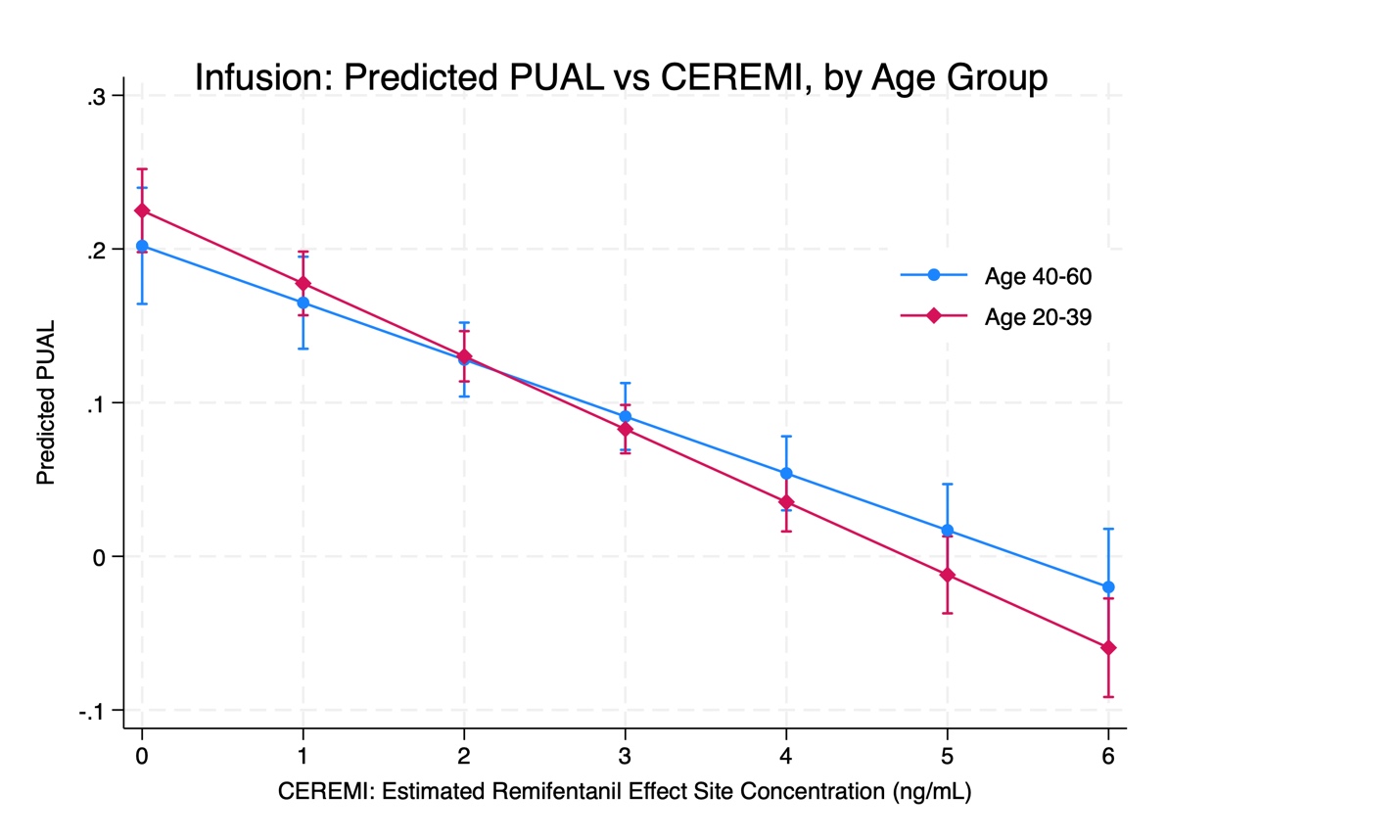


**Figure S1C.** Pupillary unrest (PUAL, mean ± 95% CI) declined consistently as modeled remifentanil effect-site concentration (CEREMI) increased during infusion, with no significant difference in slope between age groups (P = 0.125). Average predicted PUAL ± 95% CI shown.

**
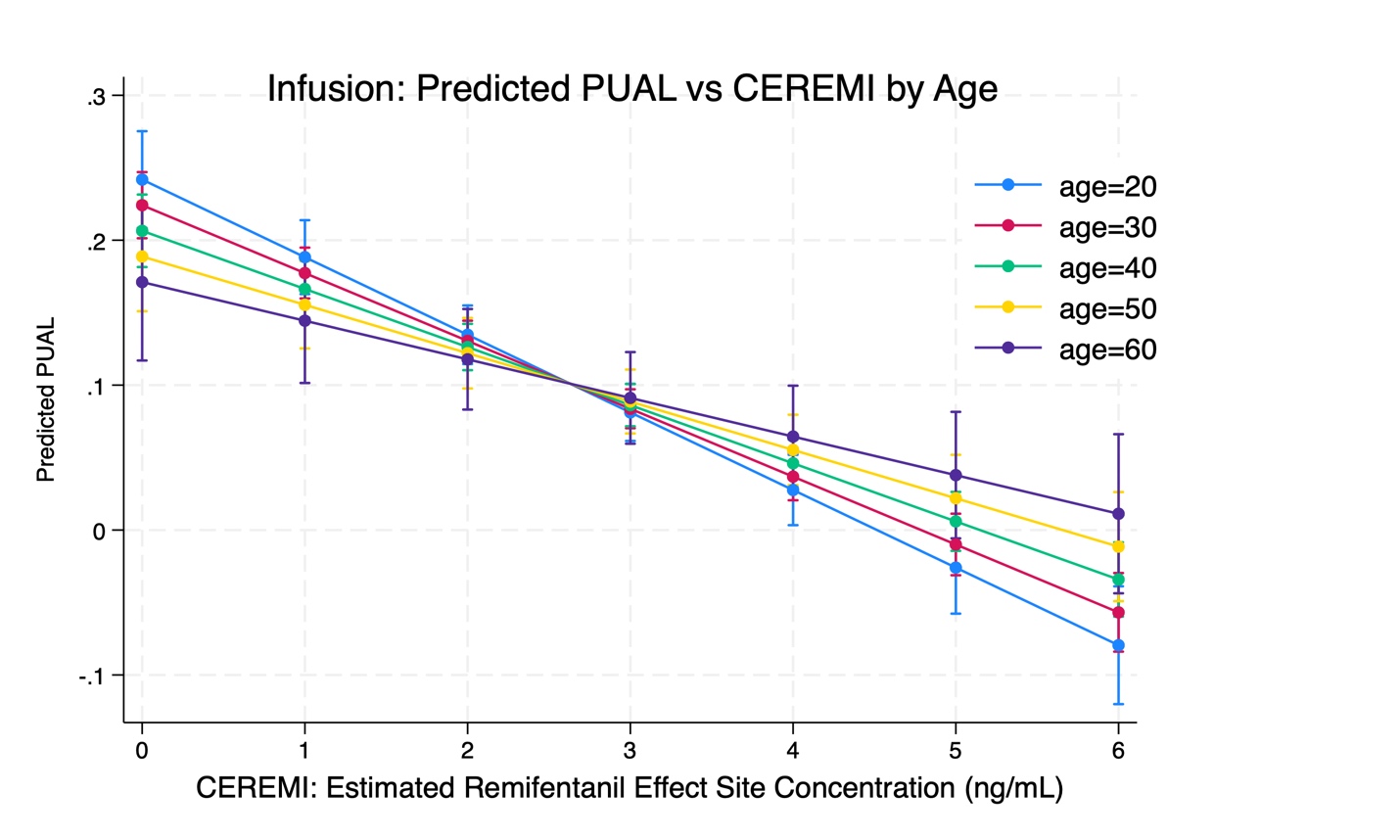
**

**Figure S1D.** Average predicted pupillary unrest (PUAL) ± 95% CI shown. PUAL declined consistently with increasing modeled remifentanil concentration (CEREMI), with the slope becoming less negative by 0.0007 units for each additional year of age (P = 0.015), flattening from –0.053 at age 20 to –0.025 at age 60.


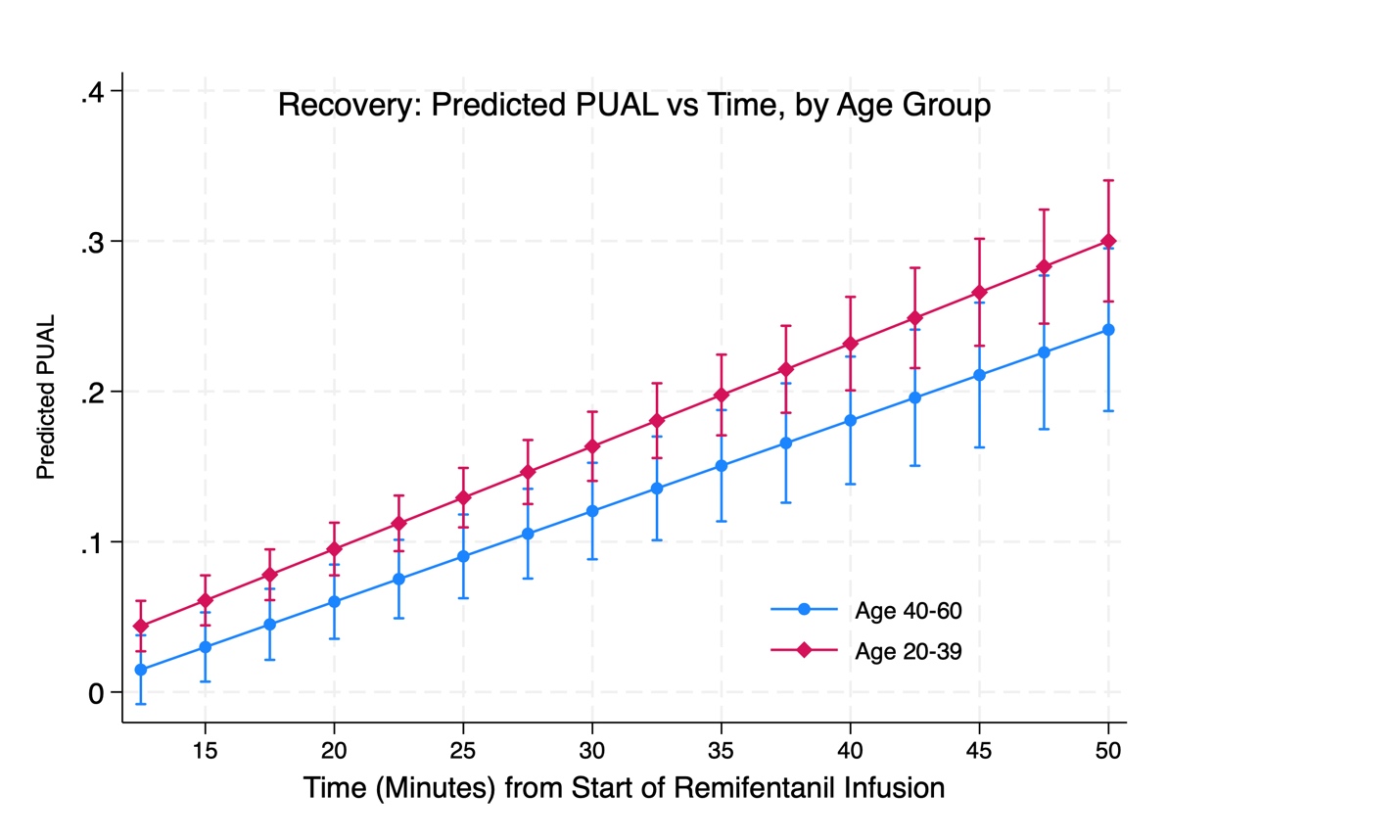


**Figure S2A.** Average predicted pupillary unrest (PUAL) ± 95% CI over time during recovery is shown. PUAL increased over time in both age groups following cessation of remifentanil, with significantly faster recovery observed in the younger group (difference in slope = 0.0023, P = 0.016).


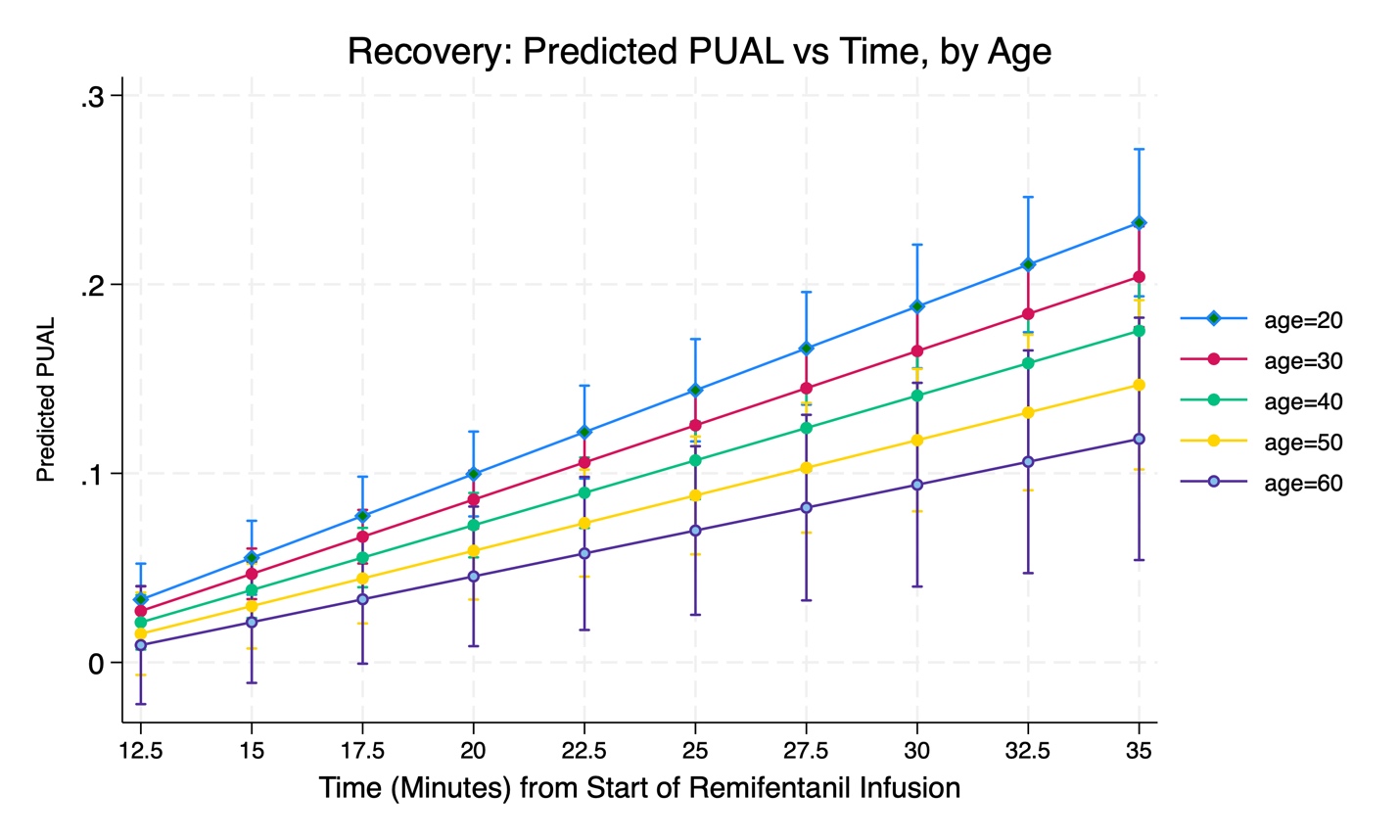


**Figure S2B.** Average predicted pupillary unrest (PUAL) ± 95% CI by age decade are shown. PUAL increased progressively across the full age range as time elapsed during recovery, with slope decreasing incrementally with advancing age (by –0.0001 per minute-year; 95% CI: –0.0002, –0.0000; P = 0.018).


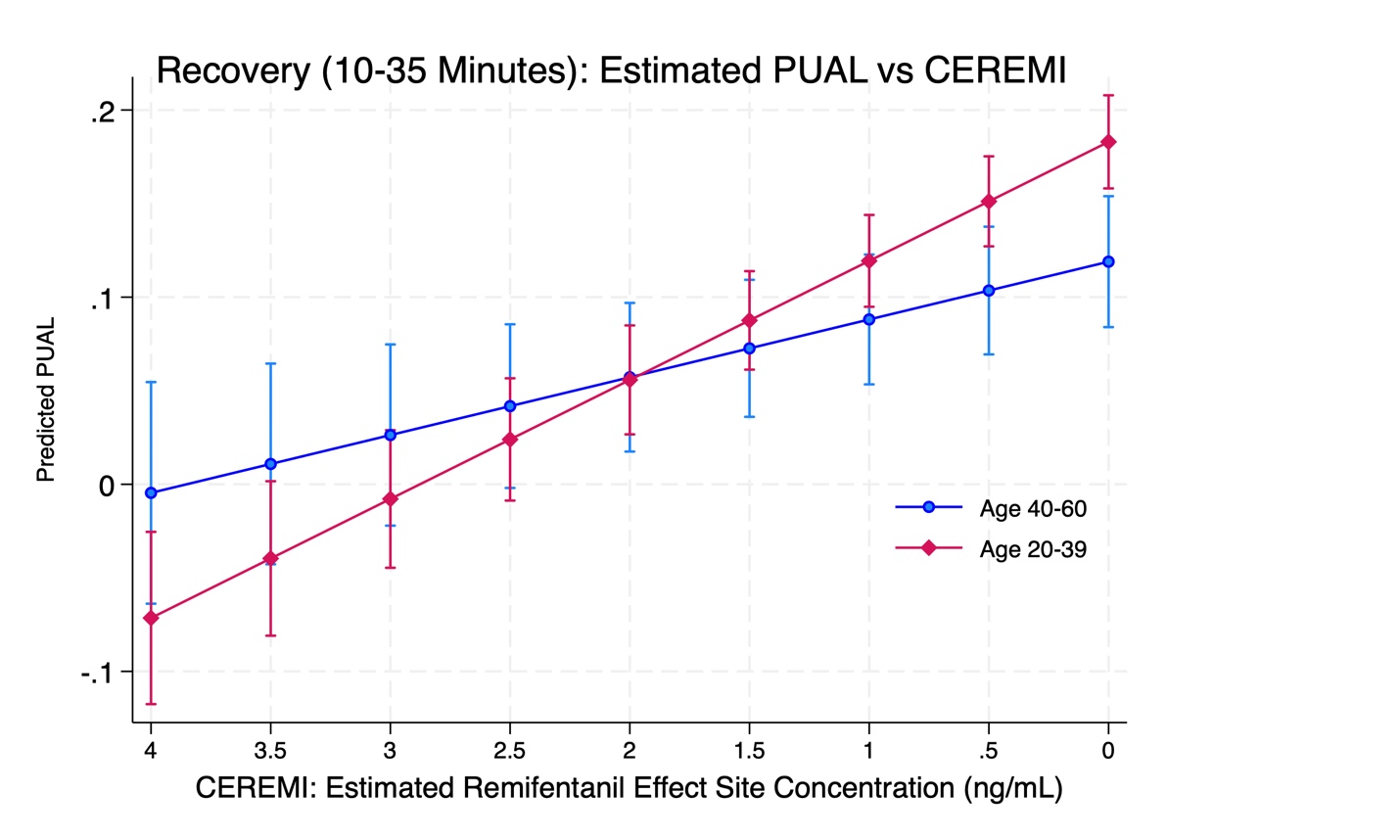


**Figure S2C.** Average predicted pupillary unrest (PUAL) ± 95% CI versus declining estimated remifentanil effect site concentration during recovery is shown. PUAL increased as modeled remifentanil concentration (CEREMI) declined, with a significantly steeper slope in younger adults (0.0636 units/ng/mL) compared to older adults (0.0309 units/ng/mL; P < 0.001 for interaction).


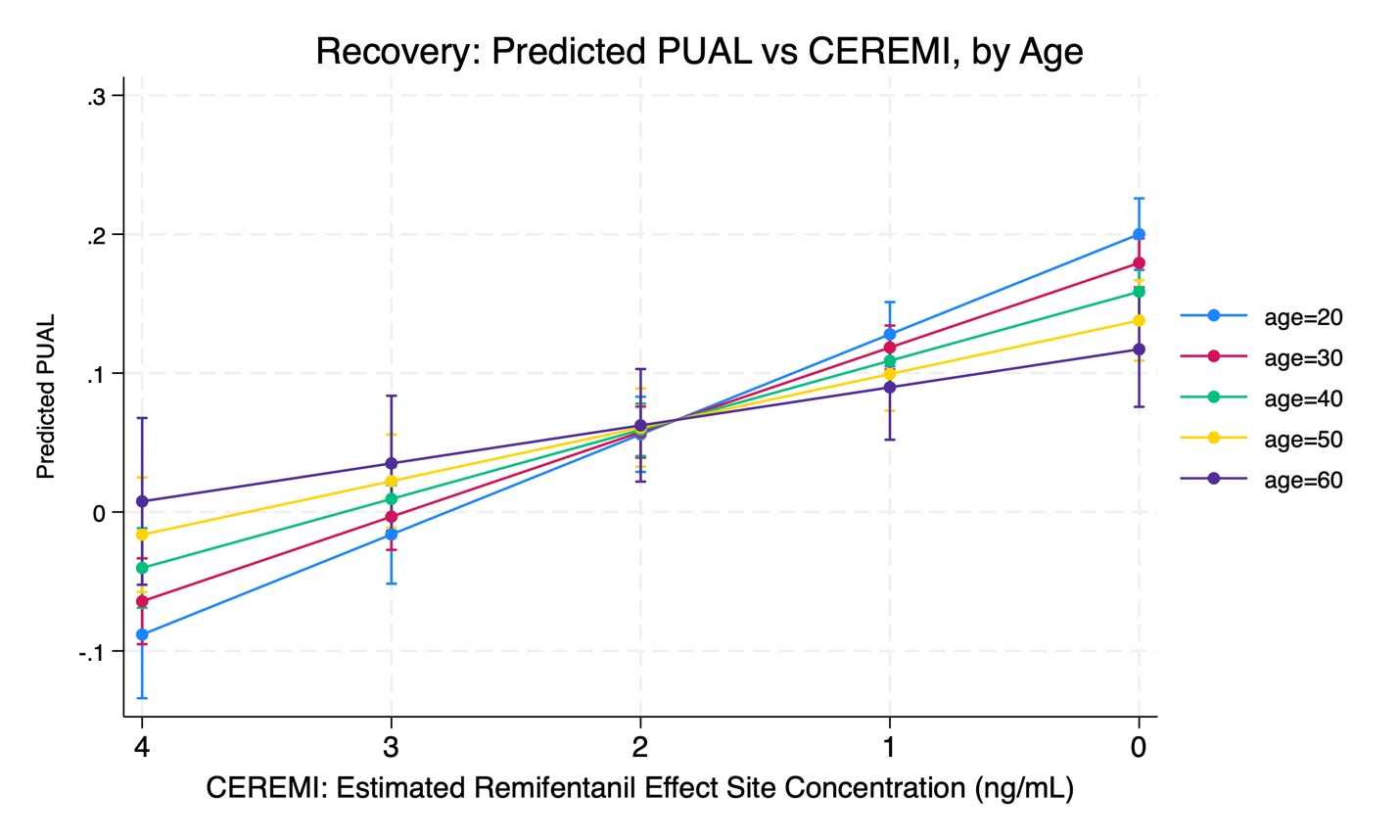


**Figure S2D.** Average predicted pupillary unrest (PUAL) ± 95% CI versus declining modeled remifentanil concentration (CEREMI) during recovery is shown. PUAL increased with declining CEREMI during recovery, with an age-dependent flattening of the slope (–0.037 + 0.0008 × age; P = 0.050), consistent with slower physiologic recovery in older individuals.

**Table S1:** Discriminative performance of pupillary unrest (PUAL) for identifying high-risk opioid exposure, defined by either time-based window (5.0–12.5-minute vs. 0–2.5-minute from start of infusion) or concentration-based thresholds (CEREMI ≥ 2.0 vs < 2.0 ng/mL).

| Risk Threshold | **AUROC (95% CI)** | **PUAL Range** | **iLR (95% CI)** |
| --- | --- | --- | --- |
|  |  |  |  |
| **Time Point from Infusion Start (Min)** | |  |  |
| **5.0 – 12.5 vs 0 – 2.5** |  |  |  |
| *Age 40-60* | **0.9700** (0.8847, 0.9959) | 0 - < 0.04 | **25.815** (1.654, 402.991) |
|  |  | ≥ 0.04 - <0.14 | 0.826 (0.450, 1.517) |
|  |  | ≥ 0.14 | 0.022 (0.012, 0.561) |
| *Age 20-39* | **0.9566** (0.9244, 0.9798) | 0 - < 0.04 | **14.591** (5.588, 38.102) |
|  |  | ≥ 0.04 - <0.14 | 0.941 (0.612, 1.445) |
|  |  | ≥ 0.14 | 0.005 (0.000, 0.075) |
| *All subjects* | **0.9586** (0.9309, 0.9791) | 0 - < 0.04 | **17.839** (6.802, 46.787) |
|  |  | ≥ 0.04 - <0.14 | 0.924 (0.641, 1.332) |
|  |  | ≥ 0.14 | 0.004 (0.000, 0.064) |
| **CEREMI ≥ 2.0 vs < 2.0 ng/mL** | |  |  |
| ***Phase: Infusion*** |  |  |  |
| *Age 40-60* | **0.9833** (0.8935, 0.9995) | 0 - < 0.04 | **27.984** (1.787, 438.327) |
|  |  | ≥ 0.04 - <0.14 | 0.754 (0.380, 1.497) |
|  |  | ≥ 0.14 | 0.030 (0.002, 0.477) |
| *Age 20-39* | **0.9549** (0.8998, 0.9889) | 0 - < 0.04 | **26.852** (3.851, 187.238) |
|  |  | ≥ 0.04 - <0.14 | 1.046 (0.555, 1.971) |
|  |  | ≥ 0.14 | 0.024 (0.003, 0.167) |
| *All subjects* | **0.9605** (0.9275, 0.9934) | 0 - < 0.04 | **40.198** (5.729, 282.074) |
|  |  | ≥ 0.04 - <0.14 | 0.908 (0.567, 1.454) |
|  |  | ≥ 0.14 | 0.017 (0.002, 0.121) |
| ***Phase: Recovery*** |  |  |  |
| *Age 40-60* | **0.8356** (0.7337, 0.9375) | 0 - < 0.04 | **3.334** (2.021, 5.500) |
|  |  | ≥ 0.04 - <0.14 | 0.466 (0.223, 0.975) |
|  |  | ≥ 0.14 | 0.115 (0.007, 1.833) |
| *Age 20-39* | **0.8985** (0.8396, 0.9574) | 0 - < 0.04 | **6.765** (3.976, 11.511) |
|  |  | ≥ 0.04 - <0.14 | 0.694 (0.377, 1.280) |
|  |  | ≥ 0.14 | 0.048 (0.003, 0.749) |
| *All subjects* | **0.8777** (0.8255, 0.9300) | 0 - < 0.04 | **5.398** (3.714, 7.844) |
|  |  | ≥ 0.04 - <0.14 | 0.592 (0.366, 0.958) |
|  |  | ≥ 0.14 | 0.031 (0.002, 0.497) |
| ***Phase: All*** |  |  |  |
| *Age 40-60* | **0.8716** (0.8158, 0.9273) | 0 - < 0.04 | **4.001** (2.510, 6.378) |
|  |  | ≥ 0.04 - <0.14 | 0.557 (0.355, 0.873) |
|  |  | ≥ 0.14 | 0.036 (0.002, 0.571) |
| *Age 20-39* | **0.9197** (0.8889, 0.9505) | 0 - < 0.04 | **7.331** (4.745, 11.324) |
|  |  | ≥ 0.04 - <0.14 | 0.680 (0.472, 0.982) |
|  |  | ≥ 0.14 | 0.126 (0.001, 0.201) |
| *All subjects* | **0.9034** (0.8757, 0.9311) | 0 - < 0.04 | **5.873** (4.262, 8.094) |
|  |  | ≥ 0.04 - <0.14 | 0.633 (0.476, 0.843) |
|  |  | ≥ 0.14 | 0.009 (0.001, 0.147) |
